# Comparative Analysis of Technologies for Quantifying Extracellular Vesicles (EVs) in Clinical Cerebrospinal Fluids (CSF)

**DOI:** 10.1371/journal.pone.0149866

**Published:** 2016-02-22

**Authors:** Johnny C. Akers, Valya Ramakrishnan, John P. Nolan, Erika Duggan, Chia-Chun Fu, Fred H. Hochberg, Clark C. Chen, Bob S. Carter

**Affiliations:** 1 Center for Theoretical and Applied Neuro-Oncology, University of California San Diego, San Diego, California, United States of America; 2 Scintillon Institute for Biomedical and Bioenergy Research, San Diego, California, United States of America; 3 Izon Science, Christchurch, New Zealand; 4 Neurology Service, Massachusetts General Hospital, and Program in Neuroscience, Harvard Medical School, Boston, Massachusetts, United States of America; University of Colorado Boulder, UNITED STATES

## Abstract

Extracellular vesicles (EVs) have emerged as a promising biomarker platform for glioblastoma patients. However, the optimal method for quantitative assessment of EVs in clinical bio-fluid remains a point of contention. Multiple high-resolution platforms for quantitative EV analysis have emerged, including methods grounded in diffraction measurement of Brownian motion (NTA), tunable resistive pulse sensing (TRPS), vesicle flow cytometry (VFC), and transmission electron microscopy (TEM). Here we compared quantitative EV assessment using cerebrospinal fluids derived from glioblastoma patients using these methods. For EVs <150 nm in diameter, NTA detected more EVs than TRPS in three of the four samples tested. VFC particle counts are consistently 2–3 fold lower than NTA and TRPS, suggesting contribution of protein aggregates or other non-lipid particles to particle count by these platforms. While TEM yield meaningful data in terms of the morphology, its particle count are consistently two orders of magnitude lower relative to counts generated by NTA and TRPS. For larger particles (>150 nm in diameter), NTA consistently detected lower number of EVs relative to TRPS. These results unveil the strength and pitfalls of each quantitative method alone for assessing EVs derived from clinical cerebrospinal fluids and suggest that thoughtful synthesis of multi-platform quantitation will be required to guide meaningful clinical investigations.

## Introduction

Extracellular Vesicles (EVs) refer to lipid membrane enclosed nano-size particles (50–2000 nm) secreted by virtually all cell types [1]. There is mounting evidence that EVs play critical roles in a number of physiologic [2, 3] and pathologic processes, including cancer [4, 5], neuro-degenerative diseases [6, 7], and hematologic disorders [8]. The available clinical data suggest that EVs secreted by aberrant cells, such as cancer cells, transgress multiple anatomic compartments and can ultimately be detected in patient bio-fluids. For instance, we previously demonstrated that analysis of cerebrospinal fluid (CSF) EV is a plausible minimally invasive platform for assessment of tumor burden and therapeutic response in brain tumor patients [9].

Qualitative assessment of EV morphology is typically performed using Transmission Electron Microscopy (TEM). However, quantitative analysis of EVs presents significant technical challenges, in large part because of their small sizes. While large EVs (>300 nm in diamter) can be analyzed by minor adjustment of conventional flow cytometry, quantitative analysis of EVs <200 nm require significant technical modification of cytometric methods [10]. One such method was previously reported by our co-author termed vesicle flow cytometry (VFC) [11]. The method involves labeling of EV by fluorescent lipid probes followed by single particle analysis using a high sensitivity flow cytometer. In addition to such specialized single particle analysis methods, two other technologies are frequently employed for quantitative assessment of EVs. The Nanoparticle tracking analysis (NTA) uses a digital camera to capture movement of EVs over a series of frames. The rate of the particle movement is then used to calculate particle size [12]. A second method, termed tunable resistive pulse sensing (TRPS), employ a two chamber set up where by the two cells are separated by a membrane containing a single pore. As EVs pass through the pore, a transient change in ionic current flow is detected and used to calculate the volume of the EV [13].

While comparative analyses of these technologies have been performed using artificially prepared beads or EVs isolated from cultured cell lines [14, 15], such comparisons have not been performed using EVs isolated from clinical specimens. Here we compared quantitative EV particle assessment using cerebrospinal fluids derived from glioblastoma patients using VFC, NTA, and TRPS.

## Materials and Methods

### Clinical Specimen Collection

All research performed were approved by IRB boards at University of California, San Diego Human Research Protections Program under IRB 120345X and were in accordance with the principles expressed at the declaration at Helsinki. Each patient was consented by a dedicated clinical research specialist prior to collection. Written consent was obtained for each patient. The consent process was approved by the ethics committee, and all records were documented in our electronic record system. The written consent from patients was also scanned into our electronic filing system. CSF was collected by ventricular/lumbar drain placement or cisternal aspiration at the time of craniotomy. Collected CSF specimens were 0.8μm filtered, snapped frozen and stored at -80°C.

### EV isolation

The EV fractions were isolated by differential centrifugation as previously described [16]. CSFs were diluted 1:1 with 1x PBS (Mediatech) prior to centrifugation. Samples were centrifuged at 2,000×g for 20 min to remove cellular debris. The supernatant was further centrifuged at 10,000×g for 30 min to pellet microvesicle. The resultant supernatant was subjected to ultracentrifugation at 120,000×g for 2 h in a Type 70 Ti rotor (Beckman) to pellet the exosomes. All centrifugation steps were performed at 4°C. EV pellets were resuspended in PBS and stored at -80°C.

### Nanoparticle tracking analysis

A Nanosight LM-10HS instrument equipped with a 405nm laser (Malvern) was calibrated with polystyrene latex microbeads at 100 nm and 200 nm prior to nanoparticle tracking analysis. Resuspended vesicles were diluted with PBS to achieve between 20–100 objects per frame. EVs were manually injected into the sample chamber at ambient temperature. Each samples was measured in triplicate at camera setting 13 with acquisition time of 30 s and detection threshold setting of 7. At least 200 completed tracks were analyzed per video. The NTA analytical software version 2.3 was used for capturing and analyzing the data.

### Tunable resistive pulse sensing

TRPS measurements were performed on an Izon qNano instrument. Data acquisition and analysis were performed using the Izon Control Suite software version V3.1. EV samples were analyzed using NP200, NP300 and NP1000 nanopores at 8 mbar pressure. Calibration runs were performed before and after each samples run using 200 nm and 950 nm diameter carboxylated polystyrene beads. Each EV sample is measured under 3 dilutions and 3 repeats for each dilution. With a bin size of 5 nm, the size and concentration distribution of each measurement is exported for average.

### Vesicle flow cytometry

EV samples were diluted to ~10^9^ nanoparticles/mL (as determined by NTA) into 100 μL of 0.1 μm filtered HEPES buffered saline (HBS; 150 mM NaCl, 10 mM HEPES pH 7.4) containing 500 nM di-8-ANEPPS, 0.01% Pluronic-127, 5 mM CaCl2 and 20 μM PPAK in a row of a 96 well plate, stained for at least 60 minutes, then diluted 1:800 in HBS and analyzed using a custom high sensitivity flow cytometer [11]. Vesicle diameter was estimated by comparison to di-8-stained liposomes (120 nm mean diameter) as described. The vesicle specificity of di-8-ANEPPS results from the fluorescence enhancement that results upon binding and insertion of the probe into the outer leaflet of a bilayer membrane. Albumin and other plasma proteins can compete with vesicles for binding by di-8-ANEPPS, however protein binding does not result in the same fluorescence enhancement that occurs when the probe inserts into a membrane.

### Transmission Electron microscopy

EVs were adhered to formvar & carbon-coated copper grids and visualized by negative staining with 0.5% uranyl acetate. Grids were viewed using a JEOL 1200EX II transmission electron microscope and photographed using a Gatan digital camera.

## Results

### Comparison of EV quantification by NTA and TRPS

**Fig 1:**
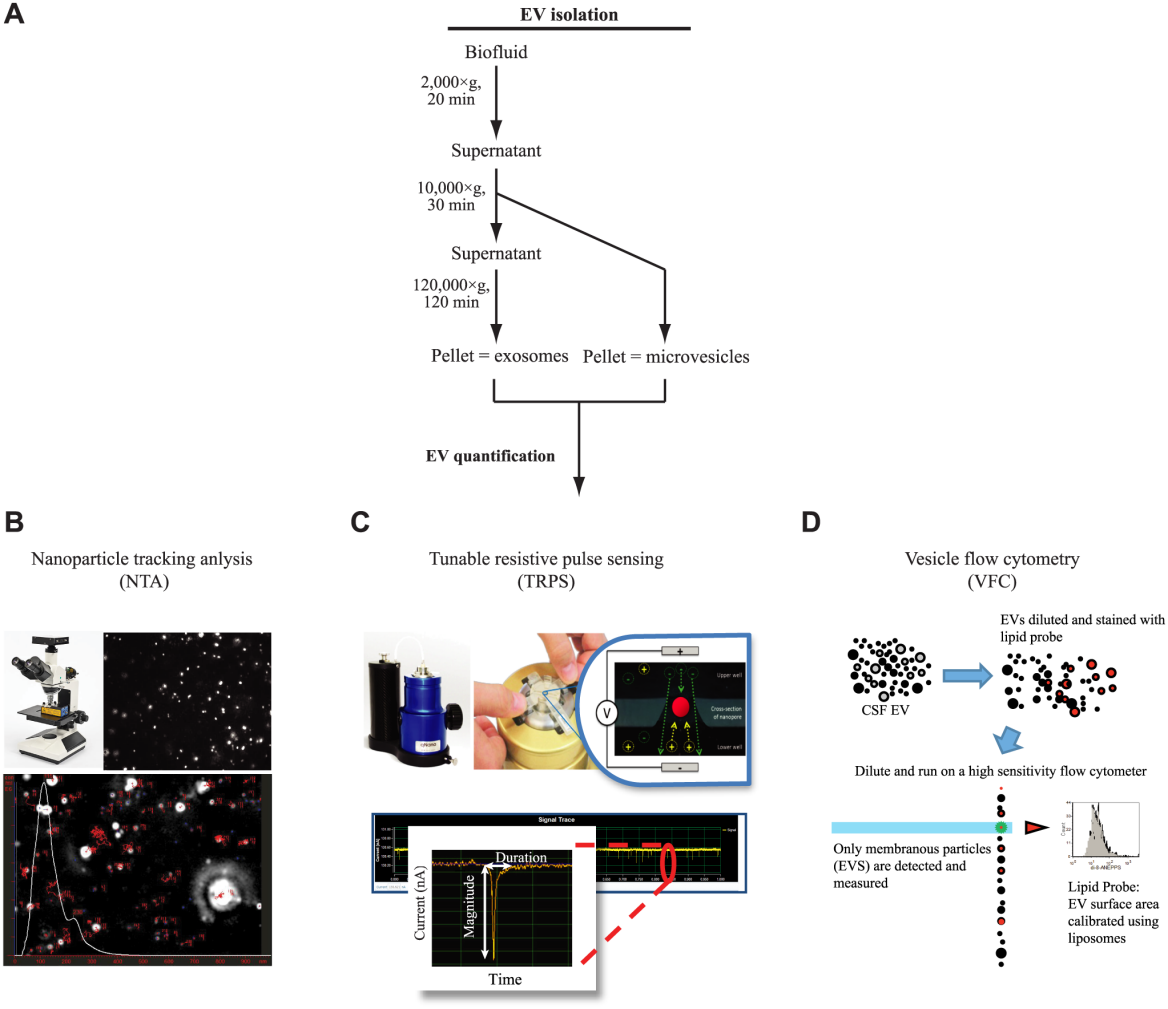
EV quantitative analysis. **(A)** Schematic representation of protocol used for the isolation of CSF microvesicles and exosomes. **(B)** In nanoparticles tracking analysis, light scattered by EVs is captured by digital camera over a series of frames. The rate of the particle movement is then used to calculate particle size using the Stokes—Einstein equation. **(C)** In tunable resistive pulse sensing, EVs change the electrical resistance as they pass through a pore-based sensor resulting in a resistive pulse signal. Signals obtained from the measurements can then be used to calculate the size, concentration and charge of each particle by correlating the signal back to a set of known standards. **(D)** In Vesicle flow cytometry, EVs were stained with an optimized concentration of a fluorogenic lipophilic probe, di-8-ANEPPS, and detected on a custom high sensitivity flow cytometer. Vesicle diameter was estimated by comparison to di-8-stained liposomes.

**Fig 2:**
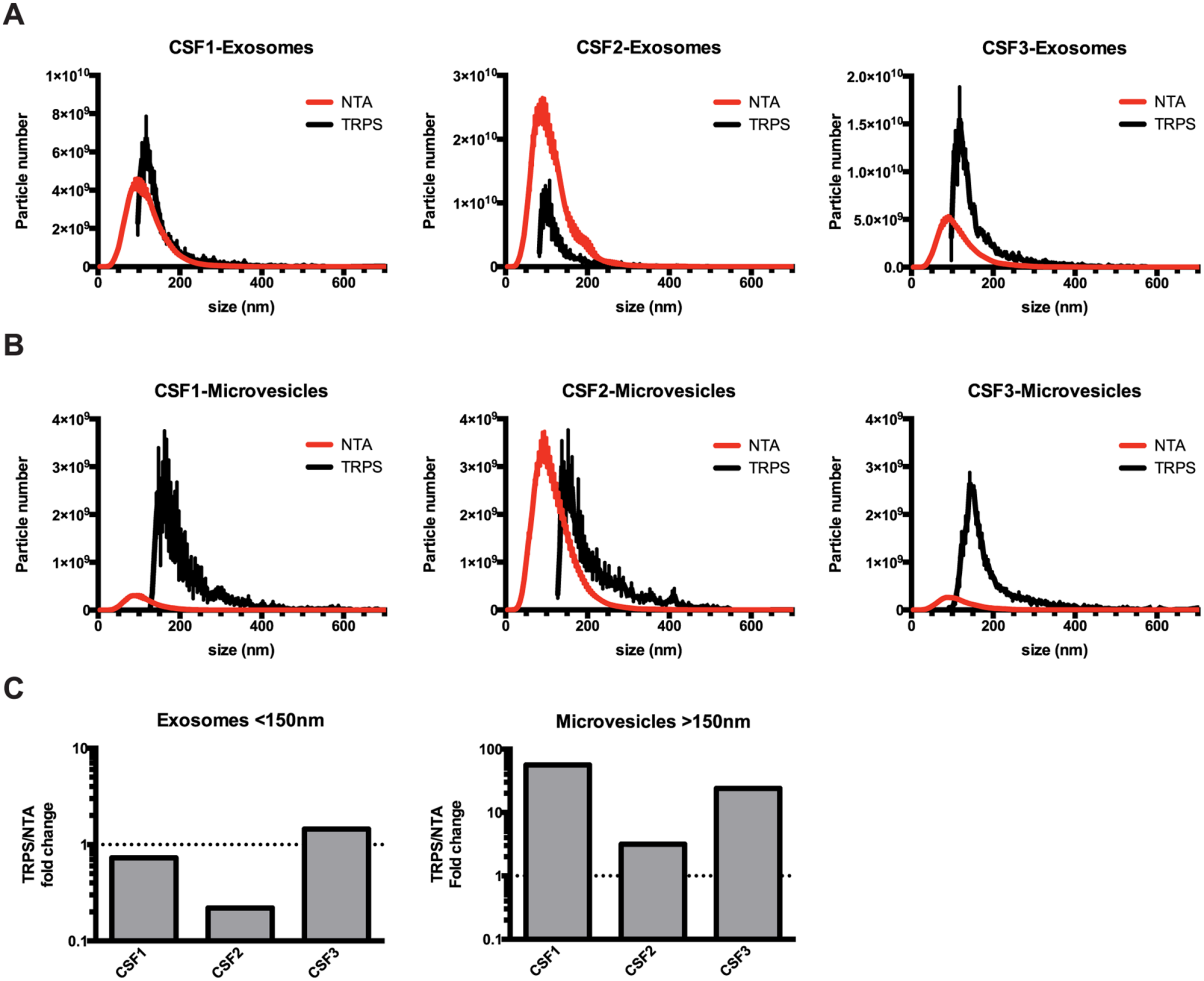
Comparison of EV quantification by NTA and TRPS. EVs were isolated from CSF collected from glioblastoma patients by differential centrifugation into microvesicle (10,000×g) and exosome (120,000×g) fractions, and resuspended in PBS. Isolated EVs were analyzed by NTA or TRPS. **(A)** Size profile of CSF exosomes determined by NTA and TRPS. **(B)** Size profile of CSF microvesicles determined by NTA and TRPS. **(C)** Comparison of EV yield by size ranges.

To evaluate the performance of the different technologies available for the characterization of extracellular vesicles derived from clinical samples, EVs were isolated from CSF by differential centrifugation into microvesicle (10,000×g) and exosome (120,000×g) fractions (Fig 1). Some smaller vesicles were pelleted at 10,000×g, leading to overlap in size profile between isolated microvesicles and exosomes. Isolated EVs were than analyzed by NTA and TRPS to determine the particle number (Table 1) and size distribution (Fig 2A and 2B). To assess the robustness of the quantitation, each sample was counted in a serial dilution (3 concentration), and each dilution was measured 3 times per instrument. We noticed good to excellent reproducibility for both methods, with NTA displaying an average of 10% error between runs and ~25% error for TRPS (Table 1). For the 120,000×g fractions, our analysis showed that NTA detected more EVs than TRPS in three of the four samples tested. However, TRPS consistently detected more particles in the 10,000×g microvesicle fractions compared to NTA (Fig 2C, Tables 1 and 2).

**Table 1:** Total EV yield as determined by NTA and TRPS.

Sample		NTA	TRPS
CSF1			
MV		5.17±0.61 × 10^9^	4.39±1.75 × 10^10^
Exo		8.43±0.68 × 10^10^	7.52±1.33 × 10^10^
CSF2			
MV		6.54±0.66 × 10^10^	4.95±1.19 × 10^10^
Exo		4.51±0.54 × 10^11^	1.08±0.34 × 10^11^
CSF3			
MV		5.10±0.42 × 10^9^	3.72±0.55 × 10^10^
Exo		9.38±0.75 × 10^10^	1.78±0.29 × 10^11^

Total EV yields are reported as mean ± SEM.

**Table 2:** EV yield by size range as determined by NTA and TRPS.

Sample		NTA	TRPS
CSF1			
MV <150 nm		4.50 × 10^9^	6.05 × 10^9^
>150 nm		6.70 × 10^8^	3.78 × 10^10^
Exo <150 nm		6.86 × 10^10^	5.04 × 10^10^
>150 nm		1.58 × 10^10^	2.48 × 10^10^
CSF2			
MV <150 nm		5.27 × 10^10^	9.35 × 10^9^
>150 nm		1.27 × 10^10^	4.01 × 10^10^
Exo <150 nm		3.77 × 10^11^	8.37 × 10^10^
>150 nm		7.46 × 10^10^	2.42 × 10^10^
CSF3			
MV <150 nm		4.10 × 10^9^	1.32 × 10^10^
>150 nm		1.00 × 10^9^	2.40 × 10^10^
Exo <150 nm		7.66 × 10^10^	1.11 × 10^11^
>150 nm		1.72 × 10^10^	6.76 × 10^10^

### Comparison of EV quantification by NTA and VFC

**Fig 3:**
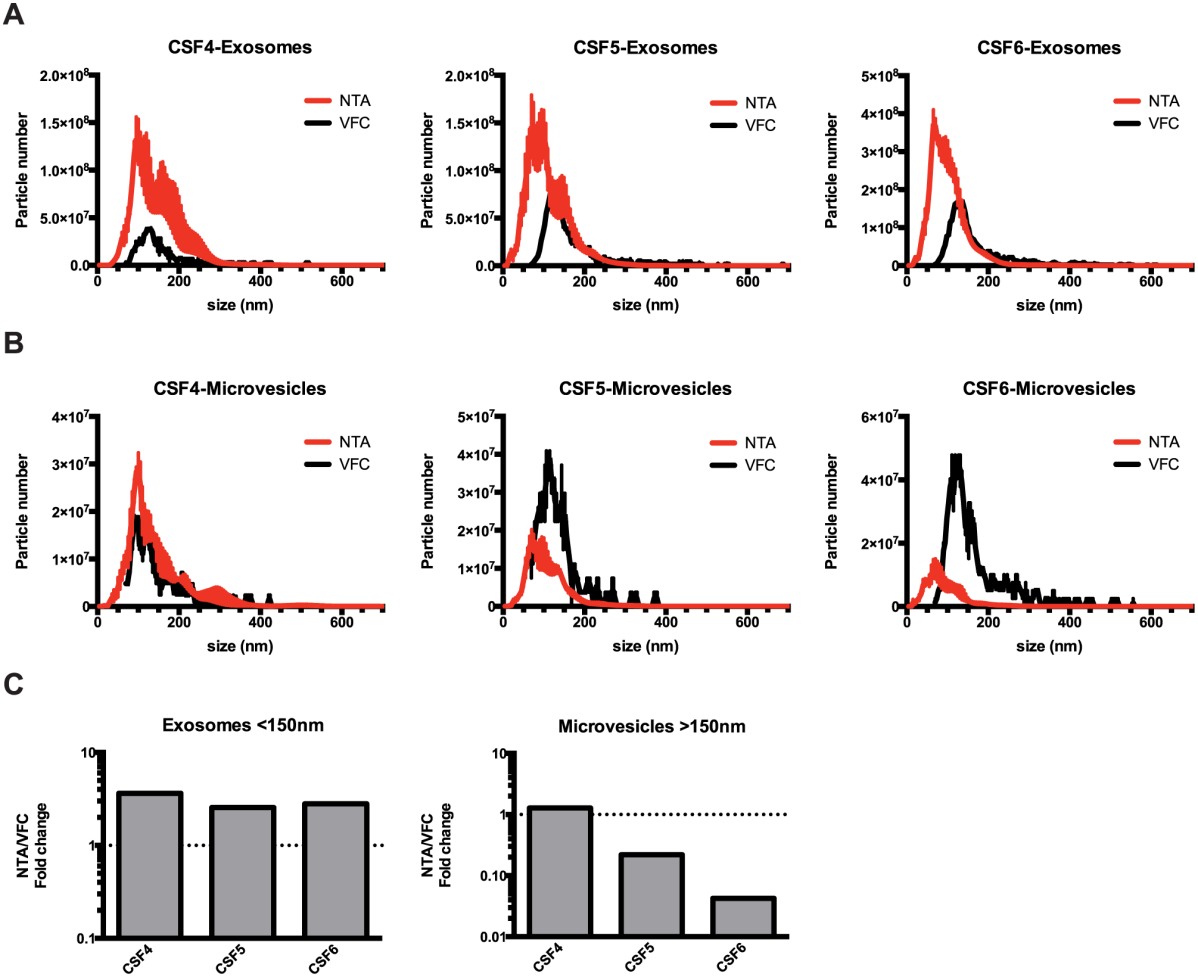
Comparison of EV quantification by NTA and VFC. CSF EVs isolated by differential centrifugation into microvesicle (10,000×g) and exosome (120,000×g) fractions were analyzed by NTA or VFC. **(A)** Size profile of CSF exosomes determined by NTA and VFC. **(B)** Size profile of CSF microvesicles determined by NTA and VFC. **(C)** Comparison of EV yield by size ranges.

While both NTA and TRPS are capable of enumerating individual nano-sized particles, neither method is capable of discriminating membranous vesicles from protein aggregates or other non-lipid particles. To more specifically detect EVs, we employed VFC, a fluorescence-triggered detection of EVs stained with a fluorogenic lipid probe. Although both NTA and VFC detected particles in similar size ranges (Fig 3A and 3B), in general, particle counts by VFC were lower than NTA (Table 3). In particular, for exosomes <150nm in diameter, NTA consistently detected more particles than VFC, suggesting that many of the small particles detected by NTA were not membranous vesicles. (Fig 3C and Table 4). However, for microvesicles >150nm in diameter, NTA detected fewer particles compared to VFC in two out of five samples. It is important to note as detection of particles by VFC relies on the incorporation of fluorogenic lipid probe into EVs, thus rendering the detection of larger vesicle with greater surface area more favorable compared to smaller vesicles.

**Table 3:** Total EV as determined by NTA and VFC.

Sample		NTA	VFC
CSF4			
MV		5.00±1.26 × 10^8^	3.68±0.83 × 10^8^
Exo		2.68±0.85 × 10^9^	7.34±1.55 × 10^8^
CSF5			
MV		2.98±0.65 × 10^8^	7.18±1.34 × 10^8^
Exo		2.82±0.68 × 10^9^	1.55±0.20 × 10^9^
CSF6			
MV		1.91±0.58 × 10^8^	9.44±1.65 × 10^8^
Exo		5.96±0.75 × 10^9^	3.37±0.32 × 10^9^

Total EV yields are reported as mean ± SEM.

**Table 4:** EV yield by size range as determined by NTA and VFC.

Sample		NTA	VFC
CSF4			
MV <150 nm		3.38 × 10^8^	2.41 × 10^8^
>150 nm		1.62 × 10^8^	1.27 × 10^8^
Exo <150 nm		1.63 × 10^9^	4.46 × 10^8^
>150 nm		1.05 × 10^9^	2.88 × 10^8^
CSF5			
MV <150 nm		2.57 × 10^8^	5.26 × 10^8^
>150 nm		4.14 × 10^7^	1.92 × 10^8^
Exo <150 nm		2.30 × 10^9^	8.98 × 10^8^
>150 nm		5.22 × 10^8^	6.56 × 10^8^
CSF6			
MV <150 nm		1.73 × 10^8^	5.32 × 10^8^
>150 nm		1.75 × 10^7^	4.12 × 10^8^
Exo <150 nm		5.37 × 10^9^	1.92 × 10^9^
>150 nm		5.90 × 10^8^	1.45 × 10^9^

### Comparison of EV quantification by NTA and TEM

**Fig 4:**
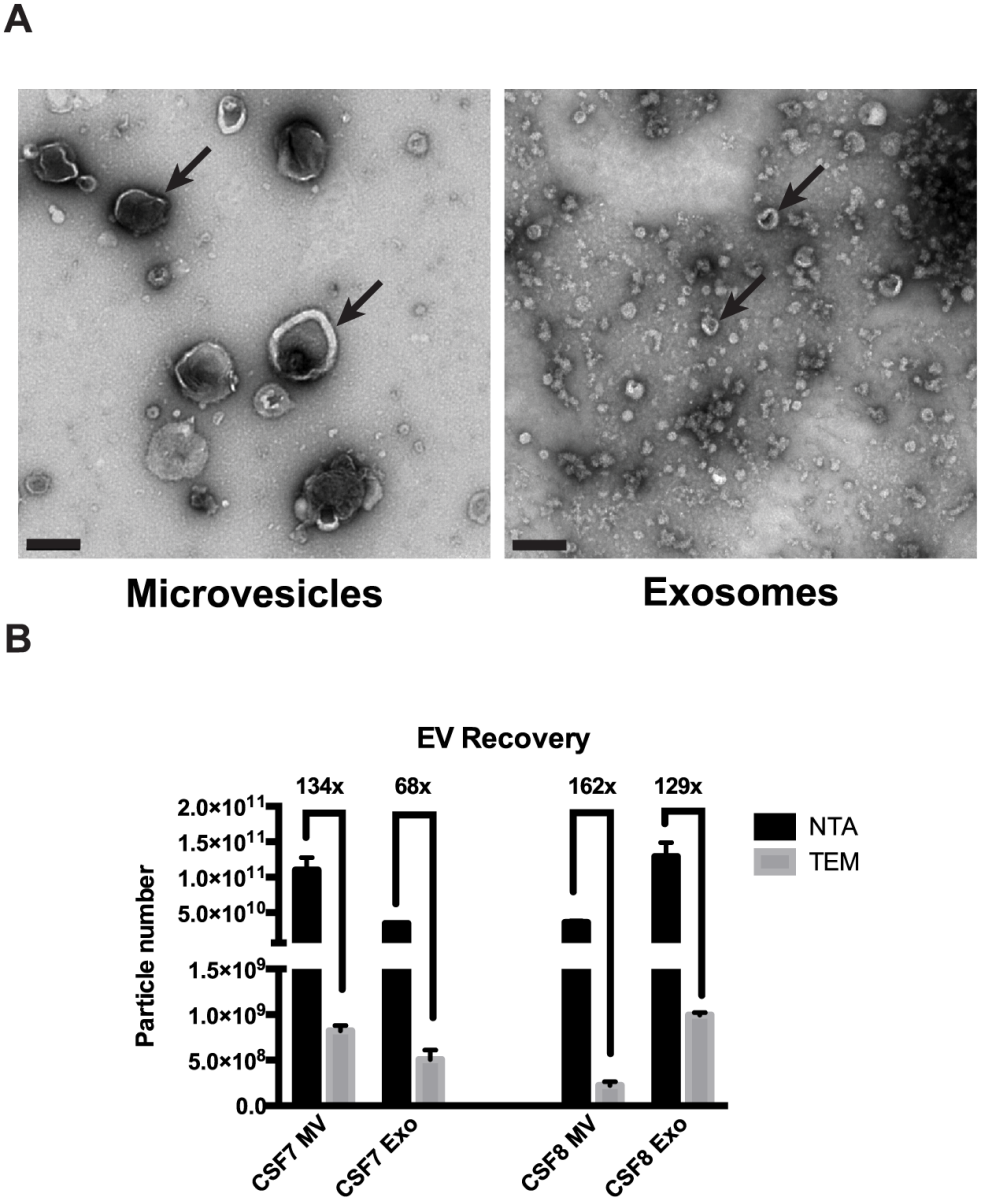
Comparison of EV quantification by NTA and TEM. CSF EVs were fractionated into microvesicles (10,000×g) and exosomes (120,000×g) by differential ultracentrifugation and then analyzed by NTA and TEM. **(A)** Representative TEM images, scale bar = 200nm. **(B)** Total EV count as determined by NTA and TEM. Fold difference in particle detected between NTA and TEM is denoted.

Finally, we evaluated TEM as a method for quantifying EVs isolated from CSF specimens (Fig 4A). CSF EVs displayed the typical “cup-shaped” morphology when visualized by TEM. EV particle counts from TEM was found to be two orders of magnitude lower than EV counts measured by NTA (Fig 4B and Table 5). This is likely due to incomplete adhesion of vesicles to the surface of the EM grids.

**Table 5:** Total EV as determined by NTA and TEM.

Sample		NTA	TEM
CSF7			
MV		1.10±0.18 × 10^11^	5.39±0.48 × 10^8^
Exo		3.47±0.11 × 10^10^	3.67±0.52 × 10^8^
CSF8			
MV		3.60±0.24 × 10^10^	1.09±0.22 × 10^8^
Exo		1.29±0.20 × 10^11^	5.59±0.46 × 10^8^

Total EV yields are reported as mean ± SEM.

## Discussion

Here we present the first comparative analysis of TEM, VFC, NTA, and TRPS in quantitative assessment of EVs isolated from clinical CSF specimens derived from brain tumor patients. At the number of EVs endogenous to this bio-fluid, all platforms generated EV concentration estimates that were highly reproducible within triplicate samples. However, we did note consistent inter-platform variations based on the size of EV. In general, TEM provide qualitative assessments of EV morphology but consistently yielded EV numbers that are two orders of magnitude lower when compared to NTA. Although NTA and TRPS yielded comparable total particle counts in the exosome fractions, NTA detected more particles in the <150 nm diameter range while TRPS detected more particles in the >150 nm diameter range. VFC particle counts are consistently 2–3 fold lower than NTA in particles <150 nm in diameter. Since VFC is performed with saturating levels of lipid probes [11], these results suggest that between 1/2 to 2/3 of NTA and TRPS scored particles were protein aggregates or other non-membranous particles. Such findings bear relevance to biomarker platforms that used the number of lipid encapsulated EVs [9] as normalizer. For larger particles (size of 150 nm or greater in diameter). NTA detected fewer numbers of particles relative to TRPS. This finding may impact quantitative assessment of EVs > 150 nm in diameter, including oncosomes [4]

An important factor that can contribute to the differences we observed in particle size distribution relates to the technology adopted for nano-particle count in each platform. With a resolving power as low as 0.2 nm, we were able to detect vesicles as small as 20 nm in diameter with TEM. However, non-uniformed adhesion of vesicles to the EM grid renders TEM sup-optimal for EV enumeration. While NTA generated particle size distribution ranging from 10 nm to 1 μm, uncertainty in the measured diffusion coefficient limits discrimination of larger sized particles [17]. Using nanopore NP200, the minimum detectable vesicle size for TRPS is ~100 nm, limiting its utility for detection of smaller EV particles. Detection of particles by VFC relies on the incorporation of fluorogenic lipid probe into EVs, which represent a subset of all extracellular particles [11]. While our study is limited in number of clinical CSF specimens examined, the theoretical benefits and limitations of these technology platforms are largely borne out in our analysis. Confirmation of these results in an expanded panel of clinical CSF specimens is warranted.

There have been two previous reports that performed comparative analysis of TRPS and NTA. One study reported comparable EV quantitation between TRPS and NTA when synthetic reference beads were assessed [14]. The second study using either artificial liposomes or EVs isolated from tissue-cultured cells documented that quantitative assessment was heavily influenced by the type of particle as well as the setting of TRPS and NTA [15]. In this regard, it is important to note that we had employed the default setting of the equipment as purchased. We reasoned that most laboratories will not have the expertise to fine-tune their purchased instruments and will use the instrument based on the default setting. We therefore felt that it is important to test the instrument in this setting. It is likely that each technology platform can be modified to enhance quantitative accuracy depending on the nature of the particles analyzed. For instance, the NTA camera level and detection threshold can be subjectively adjusted as can the membrane pore size in TRPS [15]. Similarly, In this regard, detailed methodological section should accompany results generated from such modification in order to facilitate data interpretation.

## Conclusions

At concentration of EVs present in clinical CSF isolated from glioblastoma patients, NTA and TRPS (in the default manufacturer’s settings) yielded comparable particle count while VFC particle counts are consistently 2–3 fold lower than those of NTA and TRPS. For larger particles (median sizes of 150 nm or greater in diameter), NTA reported fewer numbers of particles relative to TRPS.

## Supporting Information

S1 Fig Additional CSF samples analyzed by TRPS, NTA and VFC. **(A)** Comparison of EV yield by size ranges determined by TRPS and NTA. **(B)** Comparison of EV yield by size ranges determined by NTA and VFC.
(EPS)

S1 File Median size of EVs. CSF EVs isolated by differential centrifugation into microvesicle (10,000×g) and exosome (120,000×g) fractions were analyzed by NTA, TRPS and VFC. Table A compares the median size of EVs determined by NTA and TRPS. Table B compares the median size of EVs determined by NTA and VFC
(DOCX)
