# Supplementary material for: Comparative Analysis of Technologies for Quantifying Extracellular Vesicles (EVs) in Clinical Cerebrospinal Fluids (CSF)
Source: PLoS One. 2016 Feb 22;11(2):e0149866. doi: 10.1371/journal.pone.0149866 (PMC4763994; doi:10.1371/journal.pone.0149866)
Supplement: S1 File — CSF EVs isolated by differential centrifugation into microvesicle (10,000×g) and exosome (120,000×g) fractions were analyzed by NTA, TRPS and VFC. Table A compares the median size of EVs determined by NTA and TRPS. Table B compares the median size of EVs determined by NTA and VFC (DOCX) [file pone.0149866.s002.docx]

Table A: Median size of EVs as determined by NTA and TRPS

| **Sample** | | **NTA** | **VFC** |
| --- | --- | --- | --- |
| CSF1 | MV | 100 nm | 187 nm |
|  | Exo | 108 nm | 132 nm |
| CSF2 | MV | 101 nm | 187 nm |
|  | Exo | 106 nm | 112 nm |
| CSF3 | MV | 105 nm | 162 nm |
|  | Exo | 103 nm | 137 nm |

Table B: Median size of EVs as determined by NTA and VFC

| **Sample** | | **NTA** | **VFC** |
| --- | --- | --- | --- |
| CSF4 | MV | 120 nm | 127 nm |
|  | Exo | 131 nm | 135 nm |
| CSF5 | MV | 96 nm | 139 nm |
|  | Exo | 98 nm | 122 nm |
| CSF6 | MV | 80 nm | 139 nm |
|  | Exo | 91 nm | 143 nm |
